# Nifuratel Induces Triple-Negative Breast Cancer Cell G2/M Phase Block and Apoptosis by Regulating GADD45A

**DOI:** 10.3390/ph17101269

**Published:** 2024-09-26

**Authors:** Yuhang Hou, Hongyun Hao, Yan Yuan, Jing Zhang, Zhengrui Liu, Yimin Nie, Shichang Zhang, Shengtao Yuan, Mei Yang

**Affiliations:** 1 New Drug Screening and Pharmacodynamics Evaluation Center, National Key Laboratory for Multi-Target Natural Drugs, China Pharmaceutical University, Nanjing 210000, Chinayuany@stu.cpu.edu.cn (Y.Y.); zhangjing_991012@163.com (J.Z.); ccyfshengwu@163.com (Z.L.);; 2 Key Laboratory of Straw Comprehensive Utilization and Black Soil Conservation, Ministry of Education, College of Life Sciences, Jilin Agricultural University, Changchun 130118, China

**Keywords:** nifuratel, triple negative breast cancer, G2/M phase arrest, apoptosis, GADD45A

## Abstract

(1) Background: Nifuratel (NF113), derived from nitrofuran, has a specific anti-tumor effect. However, the potential mechanisms of NF113 in triple-negative breast cancer remain unknown. (2) Methods: In the study, CCK8 assay and colony formation assays were used to evaluate the inhibition effect of NF113 on cell proliferation. Apoptosis and cell cycle distribution were tested by flow cytometry. The mechanism of NF113’s anti-tumor effect was predicted by transcriptome sequencing and verified by using PCR and Western blot experiments. Breast cancer organoids constructed from the patient-derived tumor xenograft model and the MDA-MB-468 xenograft mouse model were established to evaluate the effect of NF113. (3) Results: Our study showed that NF113 had an anti-tumor effect on triple-negative breast cancer both in vitro and in vivo. NF113 also induced apoptosis and G2/M phase arrest in triple-negative breast cancer cells. Our experimental data further verified that NF113 reduced GADD5A mRNA and protein expression, which were significantly upregulated in breast cancer, with downstream CDC25C and AKT phosphorylation changes. (4) Conclusions: Our data provided compelling evidence that NF113 inhibited breast cancer growth via upregulating GADD45A. Conclusion: NF113 was able to exert inhibitory effects on the proliferation of triple-negative breast cancer in vivo and in vitro, which may induce G2/M phase arrest via the GADD45A/CyclinB/CDK1 pathway and apoptosis via GADD45A/JNK/P38.

## 1. Introduction

As of February 2024, the annual global incidence of cancer is nearing 20 million new cases, with approximately 9.7 million deaths per year [1]. Breast cancer, a significant type of cancer, contributes to around 2.3 million new cases and 670,000 deaths annually, accounting for 11.6% of the total global cancer incidence and 6.9% of the total cancer mortality. It ranks second in incidence and fourth in mortality among all cancer types [1]. Triple-negative breast cancer (TNBC) constitutes approximately 10% to 20% of all breast cancer cases. Despite its relatively lower prevalence, it is highly aggressive and represents the most common and invasive subtype of breast cancer, particularly among younger patients under the age of 50 [2]. TNBC is characterized by a high degree of malignancy, with a median survival time for TNBC patients typically less than 20 months, and only 9–12 months for those with advanced disease. The 5-year survival rate is less than 30% [3]. TNBC exhibits a high propensity for metastasis, with most recurrences occurring within 1–2 years. Distant metastases frequently involve visceral organs, such as the brain, liver, and lungs, while bone metastases are less common [4]. Additionally, due to the lack of benefit from endocrine therapy targeting ER and PR receptors, as well as HER2-targeted therapies, chemotherapy remains the primary treatment modality for TNBC [5,6]. However, chemotherapy is not universally effective, as only approximately one fifth of TNBC patients demonstrate a significant response, and resistance to treatment is common [7].

Nifuratel (NF113, SAP113) is a nitrofuran derivative approved in 1960 [8]. Its primary pharmacological action is to inhibit bacterial growth by interfering with enzyme systems, showing inhibitory effects against various bacteria, trichomonads, and Candida species, making it a broad-spectrum antibiotic. Clinically, it is commonly used to treat bacterial vaginosis, trichomonas vaginitis, candidal vaginitis, vulvitis, urinary tract infections, amebiasis, and giardiasis [9,10]. “Drug repurposing”, defined as identifying new clinical uses for existing drugs or candidates previously or currently undergoing preclinical or clinical research, occupies a pivotal role in pharmaceutical development [11]. Developing a completely new drug for cancer therapy typically involves multiple stages and can span over a decade, with complex processes, long timelines, and high costs [12]. In contrast, repurposing existing drugs benefits from robust preclinical and clinical data, ensuring high safety profiles. This approach can reduce development costs by approximately 40% and shorten timelines to around 3 years [13]. In conclusion, drug repurposing stands as an exceedingly effective strategy in new drug development. NF113 has been reported to induce apoptosis and cell cycle arrest in gastric cancer cells by blocking STAT3 activation, thereby inhibiting cell proliferation, survival, invasion, metastasis, and angiogenesis [14]. However, the anti-tumor effects of NF113 in vivo and the specific underlying mechanisms require further investigation, highlighting the importance and potential of deeper studies on NF113.

GADD45A, a member of the Growth Arrest and DNA Damage-inducible 45 family (which also includes GADD45B and GADD45G), is a critical stress sensor mediating various cellular responses such as DNA repair, cell cycle arrest, and apoptosis, which are significant in tumor initiation and progression [15]. GADD45A is primarily recognized for its role in maintaining genomic stability in response to DNA damage, thereby preventing tumorigenesis, which is its most extensively documented biological function [16]. Historically, the upregulation of GADD45A gene expression has been primarily identified as a downstream effect of p53 activation [17]. As a tumor suppressor protein, p53 plays a crucial role in preventing malignant transformation. Its significance extends to critical cellular processes, including DNA repair and the induction of cell cycle arrest [3]. GADD45A has been reported to induce apoptosis via pathways involving p53, p38, JNK, and NF-κB, and to regulate the cell cycle through CDK1, p21, p53, and PCNA [18]. GADD45A has also been shown to inhibit cell proliferation by directly interacting with p38, a process partly mediated by p53 [18]. Additionally, as decreased JNK enhances cell viability and allows cells to escape apoptosis, GADD45A can bind to MEKK4 and inhibit cell proliferation and induce apoptosis through the p38 and JNK pathways [19]. A recent study emphasized the role of sustained activation of the GADD45A/p21 pathway in promoting cell senescence and mitochondrial dysfunction under oxidative stress and other stressors, with p38 MAPK playing a central role in this process [20]. Therefore, GADD45A represents a potentially effective target for cancer therapy.

Breast cancer has caused significant harm to women’s health globally, making the search for more effective therapies, such as gene-targeted drugs, both necessary and urgent. These challenges underscore the urgent need for the development of novel therapeutic strategies and agents to address the unmet clinical needs in the management of TNBC. NF113, an established drug initially approved for market release in January 1960, presents a potential opportunity for repurposing in cancer treatment [8]. This study aims to evaluate the anti-tumor effects of NF113, particularly focusing on its impact on triple-negative breast cancer (TNBC). Additionally, the study seeks to explore the potential molecular targets and mechanisms through which NF113 exerts its effects on TNBC cells. By investigating these aspects, the research aims to provide insights into the viability of NF113 as a therapeutic option for TNBC, potentially contributing to the development of more targeted and effective treatments for this aggressive form of breast cancer.

## 2. Results

### 2.1. Nifuratel Has Viability Inhibition on Triple-Negative Breast Cancer Cells

**Figure 1:**
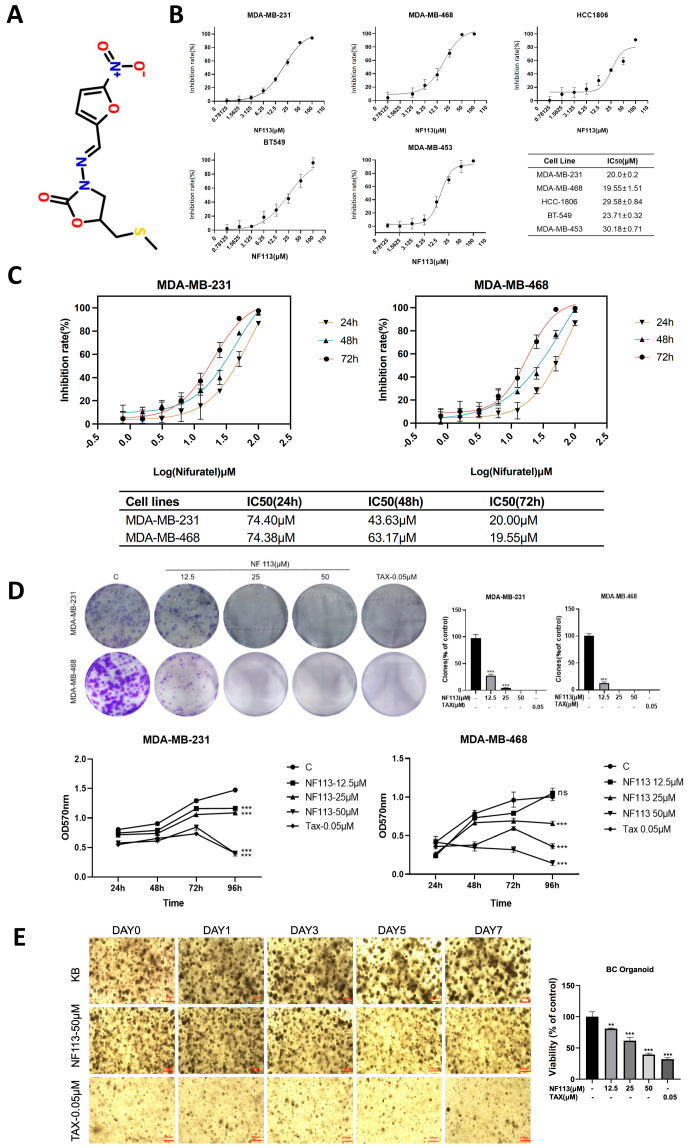
Effect of NF113 on the proliferation of triple-negative breast cancer cells. (**A**) Chemical structure of NF113. (**B**) Human TNBC cells (MDA-MB-231, MDA-MB-468, HCC-1806, BT-549, and MDA-MB-453) were treated with NF113 for 72 h. Cell viability was measured by CCK-8 assay. (**C**) MDA-MB-231 and MDA-MB-468 were treated with NF113 for 24 h, 48 h, and 72 h. Cell viability was measured by CCK-8 assay. (**D**) Effect of NF113 on Clone formation assay of MDA-MB-231 and MDA-MB-468 after treatment with NF113 or TAX (Taxol) and the growth curve of TNBC cells after NF113 treated. MDA-MB-231 and MDA-MB-468 cells were combined with NF113 (12.5, 25, 50 μM) and TAX (Taxol, 0.05 μM) cultivated together for 5 days, and the vitality value was detected using the CCK8 method. (**E**) 24 h and 48 h effect of NF113 on the proliferation of organoids. Use CCK-8 to measure changes in organoid activity after drug action. (ns, no statistical significance, ** *p* < 0.01, and *** *p* < 0.001).

Nifuratel (NF113, SAP113) is a nitrofuran derivative with the molecular formula C10H11N3O5S and a relative molecular mass of 285.27. The chemical structure of NF113 is shown in Figure 1A. Taxanes, such as paclitaxel, are commonly used as positive control drugs in anticancer research due to their well-established and potent anticancer properties. Paclitaxel, in particular, has been extensively studied and is recognized as a benchmark for evaluating the efficacy of anticancer agents. To evaluate the anti-tumor effect of NF113 on triple-negative breast cancer (TNBC) cells, we first measured the proliferation inhibition of NF113 on five different TNBC cell lines using CCK8 cell viability assays, as shown in Figure 1B. NF113 significantly suppressed the cell viability of these five TNBC cell lines—MDA-MB-231, MDA-MB-468, HCC-1806, BT-549, and MDA-MB-453—with IC50 values of 20.0 ± 0.2 μM, 19.55 ± 0.15 μM, 29.58 ± 0.84 μM, 23.71 ± 0.32 μM, and 30.18 ± 0.71 μM, respectively. As MDA-MB-231 and MDA-MB-468 were more sensitive to NF113 treatment, we choose these two cell lines to perform further experiments. The 24 h, 48 h, and 72 h IC50 results are shown in Figure 1C.

We then utilized a plate colony formation assay to evaluate the anti-proliferative effect of NF113 on TNBC cells. As shown in Figure 1D, NF113 significantly reduced the size and number of clonal clusters in MDA-MB-231 and MDA-MB-468 cells compared to the control, indicating its anti-proliferative effect. Consistently, the growth curve assessed by the CCK8 assay demonstrated that the treated group had a higher OD value compared to the control group. Furthermore, to evaluate the anti-proliferative effect of NF113 in a more clinically relevant model, we constructed breast cancer organoids derived from patient-derived xenografts (PDXO) models. NF113 inhibited the growth of breast cancer organoids in a concentration-dependent manner, as shown in Figure 1E. Collectively, these results demonstrate that NF113 effectively inhibits the growth of TNBC cells.

### 2.2. Nifuratel Induced Apoptosis of TNBC Cells

**Figure 2:**
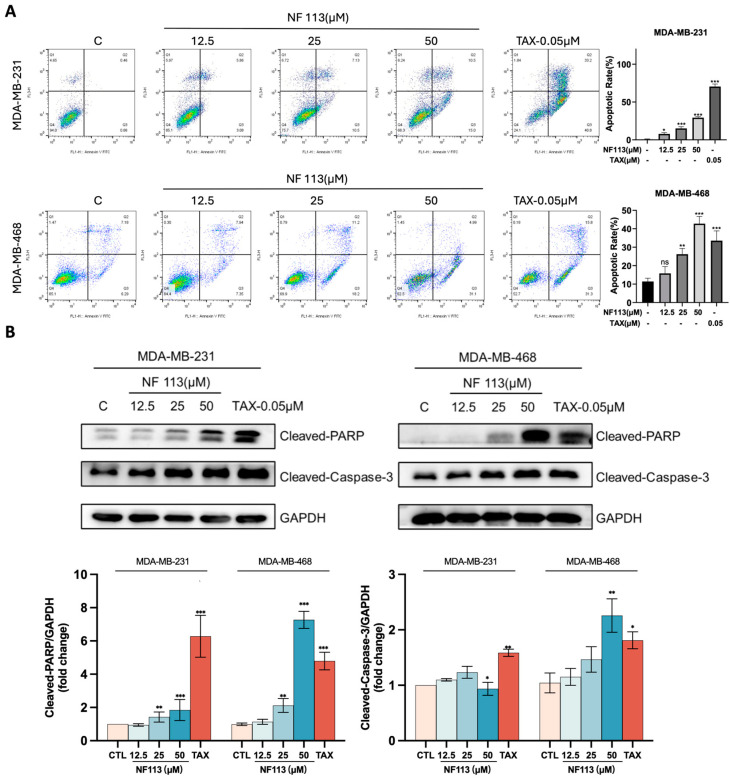
NF113 induced apoptosis of TNBC cells. (**A**) The apoptosis was detected by flow cytometry using Annexin V and PI staining methods in MDA-MB-231 and MDA-MB-468 cells after treatment with NF113 or TAX (Taxol) for 48 h. (**B**) The apoptosis marker proteins level was shown by Western Blot. Data are represented as the mean ± SD of three independent experiments. The *p*-values < 0.05 were considered statistically significant for all tests. (ns, no statistical significance, * *p* < 0.05, ** *p* < 0.01, and *** *p* < 0.001).

The anti-tumor effect is regulated through various mechanisms, including apoptosis, cell cycle arrest, and signal transduction disorder. First, we assessed apoptosis induced by NF113 using Annexin V/PI double staining and Western blot analysis on TNBC cells. As shown in Figure 2A, NF113 significantly increased apoptosis in MDA-MB-231 and MDA-MB-468 cells in a concentration-dependent manner. Consistently, Western blot results demonstrated that NF113 increased the protein levels of cleaved-PARP and cleaved-Caspase-3, which are markers of apoptosis (Figure 2B). Collectively, these results demonstrate that NF113 induces apoptosis and cell cycle arrest in TNBC cells.

### 2.3. Nifuratel Induced Phase Arrest of TNBC Cells

**Figure 3:**
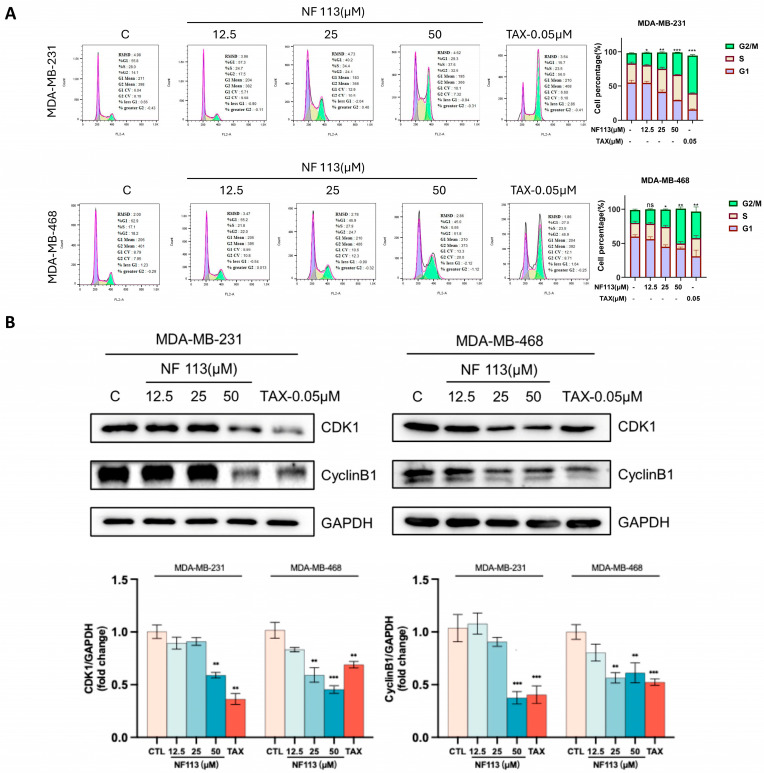
NF113 induced G2/M phase arrest of TNBC cells (**A**) Cell cycle of MDA-MB-231 and MDA-MB-468 treated with NF113 for 48 h. PI staining assay was conducted to detect the distribution of cell phase. TAX (Taxol) was used as positive control. (**B**) The G2/M phase arrest marker proteins level was detected by Western Blot. Data are represented as the mean ± SD of three independent experiments. The *p*-values < 0.05 were considered statistically significant for all tests. (ns, no statistical significance, * *p* < 0.05, ** *p* < 0.01, and *** *p* < 0.001).

To evaluate the effect of NF113 on the cell phase of TNBC cell lines MDA-MB-231 and MDA-MB-468, we performed flow cytometry assays (PI staining) and WB assays. As the results show in Figure 3A, NF113 induced G2/M phase arrest in TNBC cells, with a significant increase in the proportion of cells in the G2/M phase in NF113-treated TNBC cells. WB results further suggested this conclusion, with a concentration-dependent decrease in the proteins CDK1 and CyclinB1, which are associated with phase arrest (Figure 3B). Therefore, these results suggest that NF113 induces G2/M phase arrest in TNBC cells.

### 2.4. Nifuratel Inhibited Cell Proliferation Arrest by Increasing GADD45A Levels

**Figure 4:**
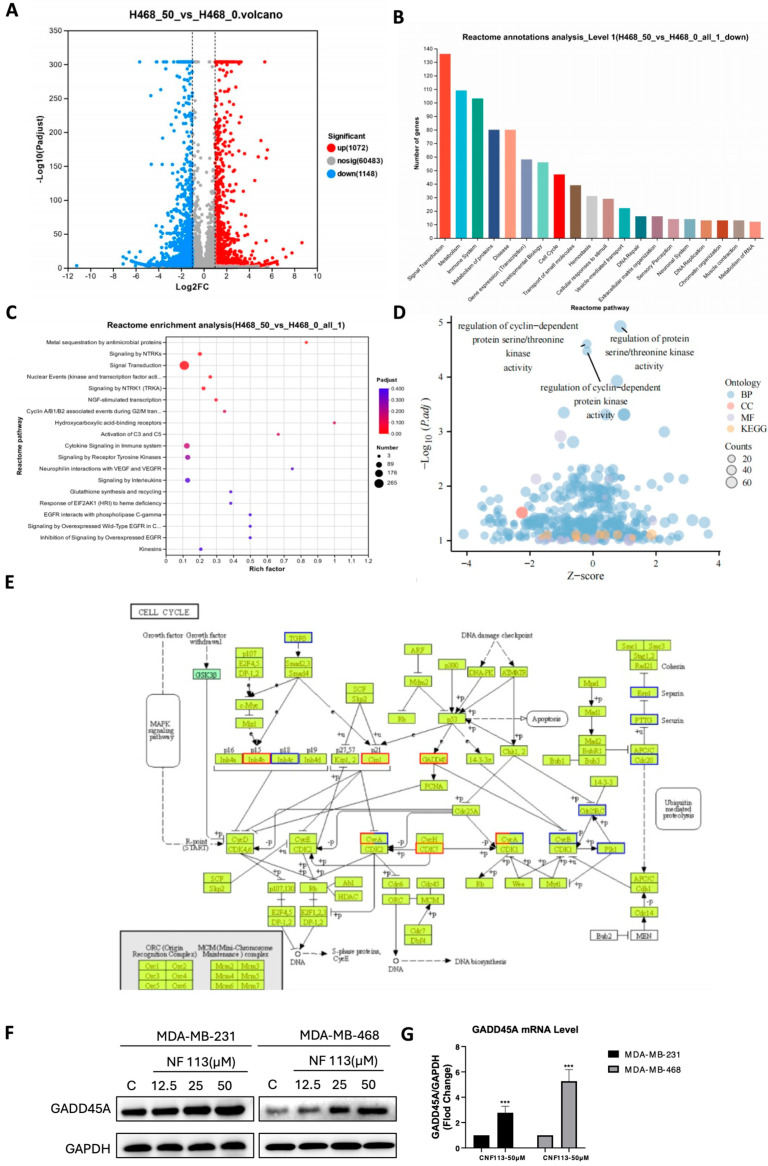
NF113 inhibited TNBC cell proliferation by upregulating the expression of GADD45A. (**A**) Volcano plot of differentially expressed genes. The blue spots are downregulated genes and red spots are upregulated genes. (**B**) GO/KEGG enrichment analysis of differentially expressed genes. (**C**) GO/KEGG combined with log_2_FC enrichment analysis of differentially expressed genes. **(D**) Enrichment analysis of differentially expressed genes in the Reactome database. (**E**) The GADD45A related signaling pathway has a red border indicating upregulation and a blue border indicating downregulation. (**F**,**G**) GADD45A protein and mRNA levels in breast cancer cells after NF113 treatment. (*** *p* < 0.001).

To explore the specific mechanisms by which NF113 exerts its anti-tumor effects on TNBC cells, we conducted transcriptomic sequencing of MDA-MB-468 cells with and without NF113 treatment. A volcano plot revealed that 1072 genes were significantly upregulated and 1148 genes were significantly downregulated after NF113 treatment compared to the control (Figure 4A). To further investigate the signaling pathways these genes may be involved in, we performed pathway enrichment analysis using GO, KEGG, and Reactome databases. GOKEGG combined analysis indicated that the top three significantly altered pathways were related to the cell cycle (Figure 4B,D). 

Next, we identified the most relevant and significantly different signaling cascades among the top-ranked differentially expressed genes from the three major databases. As shown in Figure 4E, the upregulation of GADD45A (growth arrest and DNA damage inducible 45) inhibits the expression of CyclinB, preventing the binding of CyclinB to CDK, and subsequently disrupts the CyclinB-CDK/PLK1/CDC25C loop, leading to cell cycle arrest. We next examined the expression of GADD45A of TNBC cells, and we found that GADD45A mRNA and protein levels were significantly upregulated in both MDA-MB-468 and MDA-MB-231 cells after NF113 treatment (Figure 4F,G). Thus, based on the results of transcriptomic sequencing, our finding showed that NF113 may exerts its anti-tumor effect by increasing GADD45A levels.

### 2.5. Nifuratel Regulated the Expression of GADD45A and Its Downstream Signaling Pathways

**Figure 5:**
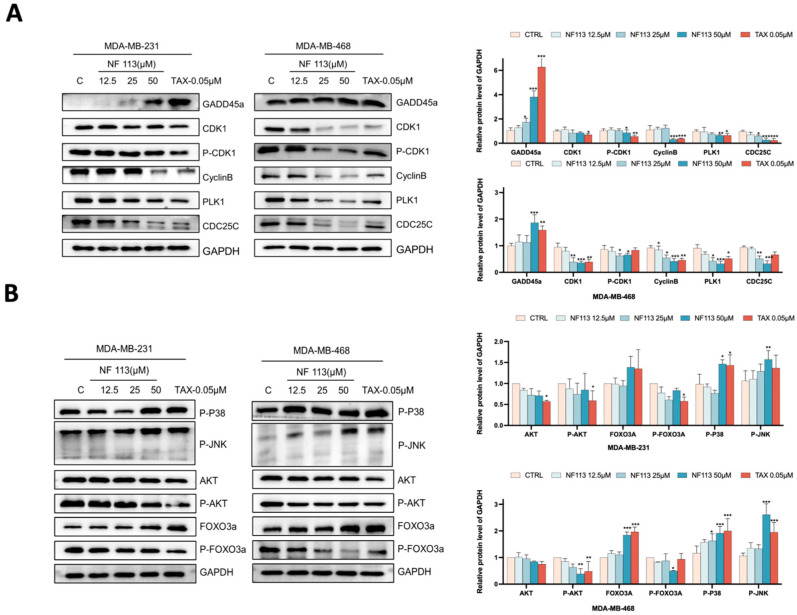
NF113 increased the expression of GADD45A and activated the downstream targets of GADD45A. (**A**) The expression levels of GADD45A, CyclinB1, CDK1, P-CDK1, and CDC25C were examined by Western Blot assay. (**B**) The expression levels of P-AKT, AKT, P-FOXO3a, FOXO3a, P-P38, and P-JNK were examined by Western Blot assay. (* *p* < 0.05, ** *p* < 0.01, and *** *p* < 0.001).

As the expression of GADD45A was significantly upregulated and the downstream signaling pathways of GADD45A are highly related to both cell apoptosis and cell phase arrest, to further investigate the precise mechanisms of NF113 on TNBC cells, we next performed WB assays to explore the downstream targets of GADD45A. As is shown in Figure 5A, the expression of CyclinB1, CDK1, p-CDK1, PLK1, and CDC25C was decreased in a concentration-dependent manner. 

Previous studies have shown that upregulated GADD45A promotes apoptosis by activating the JNK/P38 pathway through interaction with MEKK4/MTK1 [21]. As shown in Figure 5B, the levels of p-JNK and p-P38 increased in a concentration-dependent manner. Interestingly, enrichment analysis of differentially expressed genes in NF113-treated MDA-MB-468 cells using the KEGG database revealed a potential close association between NF113 and the FOXO signaling pathway. Consistently, we indicated that NF113 may inhibit AKT activity, thereby increasing FOXO3a-mediated expression. The protein level of p-AKT significantly decreased, while the protein level of AKT remained unchanged. These results suggest that NF113 activates GADD45A through the AKT/FOXO3a pathway, inducing cell cycle arrest via the GADD45A/CyclinB-CDK/PLK1/CDC25C axis and apoptosis through the JNK/P38 pathway.

### 2.6. Nifuratel Inhibited TNBC Growth In Vivo

**Figure 6:**
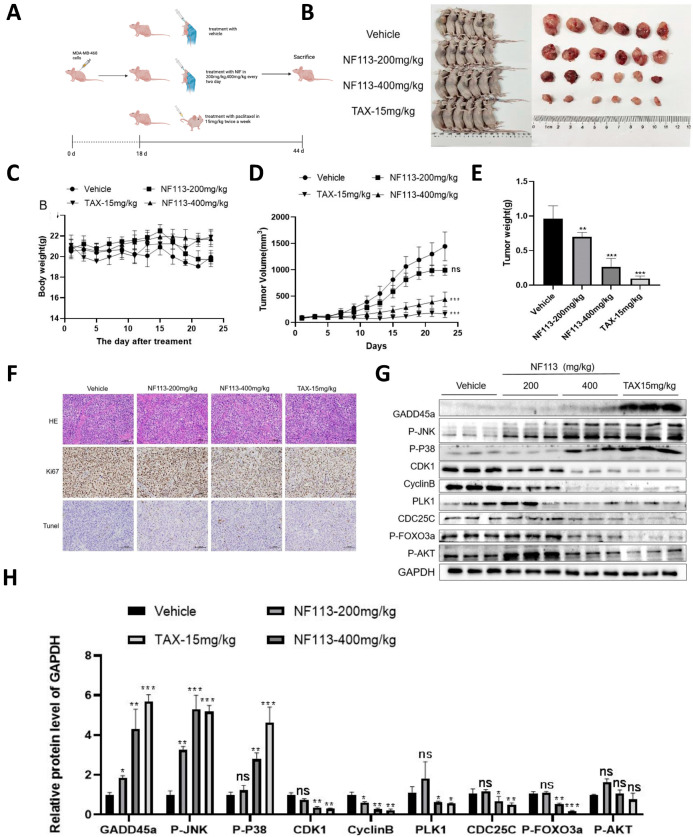
NF113 inhibited the proliferation of TNBC in vivo. (**A**) Experimental flow chart. (**B**) Schematic diagram of mice and their internal tumors. (**C**) Schematic diagram of mouse weight. (**D**) Schematic diagram of tumor volume. (**E**) Schematic diagram of tumor weight. (**F**) The images of H&E staining, Ki-67 staining, and TUNEL staining of tumor tissue of xenograft tumor model of human triple-negative breast cancer cell line, scale bar: 100 μm. (**G**,**H**) The protein expression level of tumors in the CDX model. Western blot was used to detect the expression of GADD45A, CDK1, CyclinB, PLK1, CDC25C, P-P38, P-JNK, and P-FOXO3a. GAPDH is used for load control. (ns, no statistical significance, * *p* < 0.05, ** *p* < 0.01, and *** *p* < 0.001).

We next established a cell-derived xenograft (CDX) model to further confirm the anti-tumor effect of NF113 on TNBC (Figure 6A). As shown in Figure 6B,E, NF113 significantly decreased tumor growth in tumor-bearing nude mice compared to vehicle-treated mice. The tumor volume and tumor weight were significantly lower in the NF113 -treated group (400 mg/kg) than in the vehicle-treated group. Immunohistochemical staining assays were then conducted to assess the apoptotic activity induced by NF113 in TNBC. Compared to vehicle-treated mice, NF113-treated mice (400 mg/kg) exhibited decreased Ki67 expression in tumor tissue, while TUNEL-positive cells were significantly increased, indicating that NF113 inhibits TNBC cell proliferation and induces apoptosis in vivo (Figure 6F).

Furthermore, the expression levels of GADD45A, p-JNK, and p-P38 were significantly increased, while the levels of CDK1, Cyclin B, PLK1, CDC25C, p-FOXO3a, and p-AKT were significantly decreased in the tumor tissue of the NF113-treated group compared to the vehicle-treated group, consistent with the in vitro data (Figure 6G,H). Taken together, our data demonstrate that NF113 exerts its anti-tumor effects by suppressing the AKT/FOXO3a pathway.

## 3. Discussion

Breast cancer remains a significant global health concern, with limited progress in preventing its occurrence and minimizing its impact [22]. Therefore, exploring novel therapeutic approaches is imperative. Nifuratel (NF113), a compound primarily recognized for its antibacterial properties, has emerged as a potential candidate for breast cancer treatment [23]. It has been reported that NF113 exhibits anti-tumor effects in gastric cells [14]. Our findings reveal its ability to induce apoptosis and G2/M phase arrest in TNBC cells. This effect is corroborated by our transcriptomic sequencing data, which suggests that NF113 may exert its anti-tumor effects by upregulating the expression of GADD45A.

GADD45A, a member of the Growth Arrest and DNA Damage-inducible 45 family, has been implicated in interactions with key cellular regulators such as p53 and NF-κB [24]. It was reported that GADD45A was overexpressed in pancreatic ductal adenocarcinoma and breast cancer at both mRNA level and protein level [25,26]. GADD45A-null mice exhibit a phenotype similar to that of p53-deficient mice, including genomic instability due to disruptions in G2/M cell cycle progression and impaired DNA repair [17]. Therefore, GADD45A is regarded as the link between p53-dependent DNA repair and cell cycle checkpoints, which are crucial for maintaining genomic integrity in the event of DNA damage [27]. Furthermore, several pathways related to p53 are affected by the transcriptional regulation of GADD45A. EGR1, FOXO3A, NFYA, POU2F1, and WT1 were significantly impacted by direct binding to specific motifs in the GADD45A promoter [28]. FOXO3A, which has been reported to block the cell cycle in G2/M and induce DNA repair, could be involved in the GADD45A mechanism [29]. It has been reported that GADD45A induces cell G2/M cycle arrest and inhibits cell proliferation by interacting with CDK1, thereby inducing the dissociation of the CDK1-Cyclin B1 complex, which inhibits the kinase activity of CDK1 [30]. In our study, we also found that NF113-treated cells exhibit higher levels of p-p38 and p-JNK proteins. Additionally, NF113-treated cells showed lower expression levels of CyclinB1, CDC25C, and CDK1, confirming the proposed mechanism. Our results indicate that NF113 treatment leads to a significant increase in GADD45A expression, subsequently affecting downstream signaling pathways associated with cell cycle regulation and apoptosis, like the AKT/FOXO3a pathway.

Although we have confirmed the antiproliferative efficacy of NF113 against triple-negative breast cancer (TNBC) across cellular, organoid, and in vivo xenograft models, its efficacy remains significantly lower than that of the positive control drug, TAX. The required dosage of NF113 is several times higher than that of TAX, yet its anticancer effect is still inferior. TAX is a milestone in the development of anticancer drugs, but its severe side effects cannot be overlooked. In contrast, NF113 exhibits minimal toxic reactions in acute toxicity tests in animals, with a median lethal dose (LD50) in rabbits that is 1500 times higher than its therapeutic dose, indicating a much higher safety profile compared to TAX [31]. This safety advantage has also been recognized in clinical use. Our research may provide an experimental foundation for the development of NF113 in the field of anticancer drugs. Given its low toxicity, NF113 could be utilized in clinical treatment through combination therapy with first-line chemotherapeutic agents, a strategy already validated by scientists in gastric cancer cells. Moreover, the GADD45A protein plays a crucial role in tumor proliferation, invasion, metastasis, angiogenesis, chemotherapy resistance, radiotherapy resistance, and clinical prognosis. NF113 may have the potential to impact these areas by modulating GADD45A expression.

## 4. Materials and Methods

### 4.1. Chemicals and Reagents

NF113 was provided by Jiangsu ZhengRui Pharmaceutical Technology Co., Ltd., Nanjing, China. Taxol (Paclitaxel, TAX) was purchased from Jiangsu Yangzijiang Pharmaceutical Group Co., Ltd., Taizhou, China. Cell counting kit-8 was purchased from Nanjing Vazyme Biotech Co., Ltd., Nanjing, China. Crystal violet was purchased from Nanjing Sunshine Biotechnology Co., Ltd., Nanjing, China. Cell Cycle and Apoptosis Analysis Kit was purchased from MedChemExpress, Monmouth Junction, New Jersey, NJ, USA. Cell-Light EdU Apollo488 In vitro Kit (100T) was purchased from Guangzhou RiboBio Co., Ltd., Guangzhou, China.

### 4.2. Cell Culture

MDA-MB-231, MDA-MB-468, HCC1806, BT-549, and MDA-MB-453 cells were purchased from Wuhan procell Biotech Co., Ltd., Wuhan, China. All the cells were authenticated by short tandem repeat (STR) profiling. MDA-MB-231 and BT549 were cultured in DMEM/F-12 medium (HyClone, Logan, UT, USA), MDA-MB-468 and MDA-MB-453 were cultured in DMEM medium (HyClone, Logan, UT, USA), and HCC1806 were cultured in RPMI-1640 medium (HyClone, Logan, UT, USA), supplemented with 10% fetal bovine serum (FBS; Grand Island, NY, USA), 100 U/mL penicillin, and 100 µg/mL streptomycin, in a humidified atmosphere (Meilun, Dalian, China) with 5% CO_2_ at 37 °C.

### 4.3. Cell Viability Assay

To evaluate the effect of NF113 treatment on breast cancer cell viability, a Cell Counting Kit-8 (CCK-8) assay was performed. Breast cancer cells were seeded at a density of 1500 cells per well in 96-well plates. Following a 72 h treatment with NF113, 20 µL of CCK-8 solution was added to each well. After an incubation period of 2–4 h, the absorbance was measured at 450 nm using a microplate reader. The inhibition rate was calculated using the following formula:

Inhibition rate (%) = (1 − absorbance of the treated/absorbance of the control group) × 100%. Inhibition rate (%) = (1 − absorbance of the control/absorbance of the treated group) × 100%.

### 4.4. Colony Formation Assay

The colony formation assay was conducted using a 6-well plate. A total of 1000 cells were seeded per well and allowed to incubate for 24 h. Following the incubation period, cells were treated with NF113 and other drugs for a duration of 7–14 days, with the media being refreshed every three days. Upon the colonies reaching an adequate size, the cells were fixed with 4% formaldehyde and subsequently stained with 1% crystal violet. Colony counts were determined using ImageJ software (iji154-win-java8). Data visualization and statistical analyses were performed using GraphPad Prism 8.0 software.

### 4.5. Cell Cycle Analysis

For the cell cycle assay, cells were seeded in a 6-well plate at a density of 2 × 10^5^ cells per well. Following a 12 h incubation period, cells were treated with NF113 or TAX for 48 h. The cell cycle distribution post-NF113 treatment was assessed by staining with propidium iodide (PI) and analyzed using FACS-Calibur flow cytometry.

### 4.6. Apoptosis Analysis

For the cell apoptosis assay, cells were seeded in a 6-well plate at a density of 2 × 10^5^ cells per well. After an initial 12 h incubation, cells were treated with NF113 or TAX for 48 h. Apoptosis was evaluated by staining with propidium iodide and Annexin V-FITC, followed by detection using flow cytometry (BD Biosciences, San Jose, CA, USA).

### 4.7. Immunoblotting Assay

After a 12 h incubation in a 6-well plate, cells were treated with NF113 or TAX for an additional 48 h. Subsequently, the cells were collected using RIPA buffer containing protease (100×) and phosphatase inhibitors (50×) and incubated at 4 °C for 10 min to obtain lysates. The cell lysates were centrifuged at 12,000 rpm for 15 min at 4 °C, and the protein concentration was determined using the bicinchoninic acid (BCA) assay.

Equal amounts of protein were separated via SDS-polyacrylamide gel electrophoresis and transferred to PVDF membranes. The membranes were blocked with 5% skimmed milk in TBST at 37 °C for 1 h, then incubated with primary antibodies overnight at 4 °C. This was followed by a 2 h incubation with secondary antibodies at room temperature. Primary antibodies were purchased from Cell Signaling Technology and diluted 1:1000 in TBST containing 5% BSA. Secondary antibodies, goat polyclonal anti-rabbit IgG-HRP and goat polyclonal anti-mouse IgG-HRP (Cell Signaling Technology, Danvers, MA, USA), were diluted 1:5000 in TBST.

Enhanced chemiluminescence reagents (Millipore, MA, USA) were used for detection, and the results were visualized using a Gel Doc (Hercules, CA, USA) image analyzer.

### 4.8. MDA-MB-468 Subcutaneous Tumor Model

Female Balb/c nude mice (4 weeks old), weighing between 18 and 22 g, were purchased from Beijing HuaChuang Biotechnology (Wuhan, China). Following a 1-week acclimatization period, 1 × 10^6^ MDA-MB-468 cells were subcutaneously injected into the armpit region of each mouse. Once the tumor volume reached approximately 100 mm^3^, the mice were divided into 4 groups. Mice in the LAS groups received NF113 via gavage every 2 days. TAX was administered intravenously at a concentration of 10 mg/kg twice a week, while the negative control group received an equivalent volume of physiological saline. After 27 days of NF113 administration, the mice were euthanized, and the tumor tissues were excised and analyzed. Tumor volume (TV) was calculated using the following formula: TV (mm^3^) = 1/2 × A × B2 (A, the longest diameter of the tumor; B, the shortest diameter of the tumor).

All animal care and surgical procedures were conducted in accordance with the guidelines of the Animal Care and Use Committee of China Pharmaceutical University.

### 4.9. Organoid Model

A human breast cancer lump, provided by the Affiliated Taizhou People’s Hospital of Nanjing Medical University (Taizhou, Jiangsu, China), was cut into 1–2 mm^3^ pieces. The tumor tissue was then implanted under the kidney capsule of mice, which were monitored daily until tumor formation. Tumor blocks were harvested from PDX mice, washed with PBS, and cut into 1 mm^3^ pieces. The tumor tissue was placed in a 15 mL centrifuge tube, digested with digestive fluid for 3 h, and an appropriate amount of PBS was added. The mixture was filtered through a 100 μM filter and centrifuged at 800 rpm/min for 3 min. The medium was cleaned, the supernatant discarded, and an appropriate amount of matrix glue was added for mixing. The resulting mixture was inoculated into 12-well plates, solidified at 37 °C, and cultured in breast cancer medium for further growth.

### 4.10. Statistical Analysis

All experimental data were expressed as mean ± S.D. Statistical significance was analyzed using GraphPad Prism 8.0.0 with one-way ANOVA. Statistical differences were indicated as follows: * *p* < 0.05, ** *p* < 0.01, and *** *p* < 0.001.

## 5. Conclusions

In summary, our study provides compelling evidence for the anti-tumor effects of NF113 in TNBC. By elucidating its mechanism of action, particularly through the regulation of GADD45A and associated pathways, we offer valuable insights into the therapeutic potential of NF113 as a targeted therapy for breast cancer. Further preclinical and clinical studies are warranted to fully explore the efficacy and safety of NF113 in breast cancer treatment.

## Data Availability

All relevant data are provided within the paper. The data supporting the findings of this study are available from the corresponding author upon reasonable request.

## References

[1] Bray F., Laversanne M., Sung H., Ferlay J., Siegel R.L., Soerjomataram I., Jemal A. (2024). Global Cancer Statistics 2022: GLOBOCAN Estimates of Incidence and Mortality Worldwide for 36 Cancers in 185 Countries. CA Cancer J. Clin..

[2] de Ruijter T.C., Veeck J., de Hoon J.P.J., van Engeland M., Tjan-Heijnen V.C. (2011). Characteristics of Triple-Negative Breast Cancer. J. Cancer Res. Clin. Oncol..

[3] Dent R., Trudeau M., Pritchard K.I., Hanna W.M., Kahn H.K., Sawka C.A., Lickley L.A., Rawlinson E., Sun P., Narod S.A. (2007). Triple-Negative Breast Cancer: Clinical Features and Patterns of Recurrence. Clin. Cancer Res..

[4] Huang X., Qiao Y., Brady S.W., Factor R.E., Downs-Kelly E., Farrell A., McQuerry J.A., Shrestha G., Jenkins D., Johnson W.E. (2021). Novel Temporal and Spatial Patterns of Metastatic Colonization from Breast Cancer Rapid-Autopsy Tumor Biopsies. Genome Med..

[5] Marra A., Curigliano G. (2021). Adjuvant and Neoadjuvant Treatment of Triple-Negative Breast Cancer with Chemotherapy. Cancer J..

[6] Li Y., Zhang H., Merkher Y., Chen L., Liu N., Leonov S., Chen Y. (2022). Recent Advances in Therapeutic Strategies for Triple-Negative Breast Cancer. J. Hematol. Oncol..

[7] Küng E., Fürnkranz U., Walochnik J. (2019). Chemotherapeutic Options for the Treatment of Human Trichomoniasis. Int. J. Antimicrob. Agents.

[8] Polatti F. (2012). Bacterial Vaginosis, Atopobium Vaginae and Nifuratel. Curr. Clin. Pharmacol..

[9] Evans B.A., Catterall R.D. (1970). Nifuratel Compared with Metronidazole in the Treatment of Trichomonal Vaginitis. Br. Med. J..

[10] Pushpakom S., Iorio F., Eyers P.A., Escott K.J., Hopper S., Wells A., Doig A., Guilliams T., Latimer J., McNamee C. (2019). Drug Repurposing: Progress, Challenges and Recommendations. Nat. Rev. Drug Discov..

[11] Sleire L., Førde H.E., Netland I.A., Leiss L., Skeie B.S., Enger P.Ø. (2017). Drug Repurposing in Cancer. Pharmacol. Res..

[12] Pantziarka P., Bouche G., Meheus L., Sukhatme V., Sukhatme V.P., Vikas P. (2014). The Repurposing Drugs in Oncology (ReDO) Project. Ecancermedicalscience.

[13] Zheng H., Hong H., Zhang L., Cai X., Hu M., Cai Y., Zhou B., Lin J., Zhao C., Hu W. (2017). Nifuratel, a Novel STAT3 Inhibitor with Potent Activity against Human Gastric Cancer Cells. Cancer Manag. Res..

[14] Patel K., Murray M.G., Whelan K.A. (2022). Roles for GADD45 in Development and Cancer. Adv. Exp. Med. Biol..

[15] Pietrasik S., Zajac G., Morawiec J., Soszynski M., Fila M., Blasiak J. (2020). Interplay between BRCA1 and GADD45A and Its Potential for Nucleotide Excision Repair in Breast Cancer Pathogenesis. Int. J. Mol. Sci..

[16] Hollander M.C., Sheikh M.S., Bulavin D.V., Lundgren K., Augeri-Henmueller L., Shehee R., Molinaro T.A., Kim K.E., Tolosa E., Ashwell J.D. (1999). Genomic Instability in Gadd45a-Deficient Mice. Nat. Genet..

[17] Tamura R.E., de Vasconcellos J.F., Sarkar D., Libermann T.A., Fisher P.B., Zerbini L.F. (2012). GADD45 Proteins: Central Players in Tumorigenesis. Curr. Mol. Med..

[18] Salvador J.M., Brown-Clay J.D., Fornace A.J. (2013). Gadd45 in Stress Signaling, Cell Cycle Control, and Apoptosis. Adv. Exp. Med. Biol..

[19] Camilleri-Robles C., Serras F., Corominas M. (2019). Role of D-GADD45 in JNK-Dependent Apoptosis and Regeneration in Drosophila. Genes.

[20] Palomer X., Salvador J.M., Griñán-Ferré C., Barroso E., Pallàs M., Vázquez-Carrera M. (2024). GADD45A: With or without You. Med. Res. Rev..

[21] Takekawa M., Saito H. (1998). A Family of Stress-Inducible GADD45-like Proteins Mediate Activation of the Stress-Responsive MTK1/MEKK4 MAPKKK. Cell.

[22] Libson S., Lippman M. (2014). A Review of Clinical Aspects of Breast Cancer. Int. Rev. Psychiatry.

[23] Zheng H., Chen Z., Cai A., Lin X., Jiang X., Zhou B., Wang J., Yao Q., Chen R., Kou L. (2020). Nanoparticle Mediated Codelivery of Nifuratel and Doxorubicin for Synergistic Anticancer Therapy through STAT3 Inhibition. Colloids Surf. B Biointerfaces.

[24] Jang H.-J., Yang J.H., Hong E., Jo E., Lee S., Lee S., Choi J.S., Yoo H.S., Kang H. (2021). Chelidonine Induces Apoptosis via GADD45a-P53 Regulation in Human Pancreatic Cancer Cells. Integr. Cancer Ther..

[25] Schneider G., Weber A., Zechner U., Oswald F., Friess H.M., Schmid R.M., Liptay S. (2006). GADD45alpha Is Highly Expressed in Pancreatic Ductal Adenocarcinoma Cells and Required for Tumor Cell Viability. Int. J. Cancer.

[26] Yu K.-D., Di G.-H., Li W.-F., Rao N.-Y., Fan L., Yuan W.-T., Hu Z., Wu J., Shen Z.-Z., Huang W. (2010). Genetic Contribution of GADD45A to Susceptibility to Sporadic and Non-BRCA1/2 Familial Breast Cancers: A Systematic Evaluation in Chinese Populations. Breast Cancer Res. Treat..

[27] Xie L., Jia L., Qu J., Chen D., Lv Y., Li H., Zheng J. (2020). Expression and Prognostic Significance of the P53-Related DNA Damage Repair Proteins Checkpoint Kinase 1 (CHK1) and Growth Arrest and DNA-Damage-Inducible 45 Alpha (GADD45A) in Human Oral Squamous Cell Carcinoma. Eur. J. Oral Sci..

[28] Takahashi S., Saito S., Ohtani N., Sakai T. (2001). Involvement of the Oct-1 Regulatory Element of the Gadd45 Promoter in the P53-Independent Response to Ultraviolet Irradiation. Cancer Res..

[29] Qi L., Wang Y., Su S., Wang M., Jablonska E., Jia Y., Wang R., Hao S., Feng C., Li G. (2022). Sodium Selenite Inhibits Cervical Cancer Growth via ROS Mediated AMPK/FOXO3a/GADD45a Axis. Chem. Biol. Interact..

[30] Zhan Q., Antinore M.J., Wang X.W., Carrier F., Smith M.L., Harris C.C., Fornace A.J. (1999). Association with Cdc2 and Inhibition of Cdc2/Cyclin B1 Kinase Activity by the P53-Regulated Protein Gadd45. Oncogene.

[31] Mendling W., Mailland F. (2002). Microbiological and Pharmaco-Toxicological Profile of Nifuratel and Its Favourable Risk/Benefit Ratio for the Treatment of Vulvo-Vaginal Infections. A Review. Arzneimittelforschung.

